# Effects of Lipid Lowering Therapies on Vulnerable Plaque Features: An Updated Narrative Review of the Literature

**DOI:** 10.3390/jcdd10060260

**Published:** 2023-06-15

**Authors:** Flavio Giuseppe Biccirè, Laura Gatto, Ylenia La Porta, Pasquale Pignatelli, Francesco Prati, Daniele Pastori

**Affiliations:** 1 Department of General and Specialized Surgery “Paride Stefanini”, Sapienza University of Rome, 00185 Rome, Italy; flaviogiuseppe.biccire@uniroma1.it; 2 Centro per la Lotta Contro L’Infarto—CLI Foundation, 00182 Rome, Italy; 3 Department of Cardiovascular Sciences, San Giovanni Hospital, 00184 Rome, Italy; 4 Department of Medicine, Campus Bio-Medical University, 00128 Rome, Italy; 5 Department of Clinical Internal, Anesthesiological, and Cardiovascular Sciences, Sapienza University of Rome, 00185 Rome, Italy; 6 Saint Camillus International Medical University, 00131 Rome, Italy

**Keywords:** lipid lowering therapy, plaque regression, plaque stabilization, optical coherence tomography, intravascular ultrasound, vulnerable plaque

## Abstract

The clinical evidence on the efficacy of lipid lowering therapy in patients with coronary artery disease (CAD) is unequivocally established. However, the effects of these therapies on plaque composition and stability are less clear. The use of intracoronary imaging (ICI) technologies has emerged as a complement to conventional angiography to further characterize plaque morphology and detect high-risk plaque features related to cardiovascular events. Along with clinical outcomes studies, parallel imaging trials employing serial evaluations with intravascular ultrasound (IVUS) have shown that pharmacological treatment has the capacity to either slow disease progression or promote plaque regression, depending on the degree of lipid lowering achieved. Subsequently, the introduction of high-intensity lipid lowering therapy led to much lower levels of low-density lipoprotein cholesterol (LDL-C) levels than achieved in the past, resulting in greater clinical benefit. However, the degree of atheroma regression showed in concomitant imaging trials appeared more modest as compared to the magnitude of clinical benefit accrued from high-intensity statin therapy. Recently, new randomized trials have investigated the additional effects of achieving very low levels of LDL-C on high-risk plaque features—such as fibrous cap thickness and large lipid accumulation—beyond its size. This paper provides an overview of the currently available evidence of the effects of moderate to high-intensity lipid lowering therapy on high-risk plaque features as assessed by different ICI modalities, reviews data supporting the use of these trials, and analyse the future perspectives in this field.

## 1. Introduction

Despite recent advances in medical and interventional therapies, coronary artery disease (CAD) continues to be a major cause of morbidity and mortality throughout the world [1].

Typically, patients present with acute coronary syndromes (ACS) defined by acute myocardial infarction (MI) or unstable angina and, if they survive the index event, they experience a non-negligible rate of subsequent cardiovascular events [2,3]. Robust data have shown that most of these future events arise from untreated atherosclerotic lesions that were non-obstructive at the time of the index coronary angiography but harbour high-risk characteristics—the so-called vulnerable plaques.

In this context, reducing plasma low-density lipoprotein cholesterol (LDL-C) levels has always been considered the cornerstone of treatment aimed at reducing the burden of coronary atherosclerosis [4]. The rationale for this approach is based upon the concept that intra-plaque accumulation of oxidized cholesterol represents by far the most important factor in the development and destabilization of coronary atherosclerotic plaques and is correlated with elevated circulating serum levels of LDL-C [5,6,7].

The first drugs proven to reduce major cardiovascular events in patients with CAD were the HMG-CoA reductase inhibitors, namely statins [8]. The use of potent statins in combination with cholesterol absorption inhibitors (i.e., ezetimibe) led to an additional clinical benefit for each 1.0 mmol/L reduction in LDL-C achieved [9,10]. More recently, the addition of proprotein convertase subtilisin kexin type 9 (PCSK9) inhibitors to statin therapy allowed to reach much lower levels of LDL-C than achieved in the past, resulting in greater clinical benefit [11,12].

Along with clinical studies, landmark imaging trials have investigated the pathophysiological mechanisms inherent to the clinical benefits of lipid lowering therapy.

Depending on the degree of lowering of LDL-C levels, intravascular ultrasound (IVUS) studies have shown that lipid-lowering therapy can not only slow disease progression but also induce a reduction in atheroma volume [13,14,15], with a linear relationship between on-treatment LDL-C levels and reduction in atheroma burden [13,16,17]. However, the overall magnitude of atheroma volume reduction obtained in IVUS trials investigating anti-atherosclerotic effects of PCSK9 inhibitors appeared modest in relation to the net clinical benefit showed in the outcomes of clinical trials achieving similar on-treatment LDL-C levels, suggesting additional vascular effects beyond the reduction in the atheroma volume [11,12,14]. Recent imaging trials employed new near-infrared spectroscopy (NIRS)-IVUS and optical coherence tomography (OCT) to solve this conundrum and reported favourably effects of potent lipid lowering therapy on plaque composition (reduction in lipid component and increase in fibrous cap thickness) beyond atheroma reduction [18].

Therefore, increasingly aggressive pharmacological therapies and new imaging modalities allowed us to move from the concept of slowing the progression of coronary atheroma, to that of regression and stabilization of coronary plaques.

In this narrative review, we summarize current evidence on the effects of moderate to high-intensity lipid lowering therapy on vulnerable plaque features, with a particular emphasis on the concepts of plaque regression and stabilization, and future perspectives.

## 2. Intracoronary Imaging and In Vivo Plaque Evaluation

Coronary angiography has historically served as the gold standard for the diagnosis of coronary artery disease. Landmark studies using stenosis diameter at invasive coronary angiography were among the first to demonstrate the possibility of disease regression in patients treated with lipid-lowering therapy [19].

More recently, the use of contemporary intracoronary imaging technologies has emerged as a complement to conventional angiography to further characterize plaque morphology.

Over the years, different intravascular imaging techniques have been introduced to investigate the extension and composition of coronary atherosclerotic plaque. In large prospective studies, intracoronary imaging (ICI) modalities were able to translate concepts emerging from ex vivo pathological studies into clinical practice and reveal the natural history of human coronary atherosclerosis [20]. These modalities have identified in vivo plaque morphological features associated with coronary events, namely “vulnerable plaque” features [21,22,23,24].

In parallel, studies employing serial ICI evaluations have been instrumental in assessing qualitative and quantitative changes induced by pharmacological therapy in patients with CAD.

The IVUS technique was the first one introduced in clinical practice, about 30 years ago, and it is based on acoustic sound wave backscattering, providing a grayscale imaging with an axial resolution of 80–120 μm and a penetration depth of 4–8 mm [20]. Thus, with its high penetration depth, IVUS enables tomographic imaging of coronary vessel wall with a high-quality quantification of the burden of the atheroma [20]. IVUS serial evaluations, performed at baseline and follow-up, have been crucial to assess for the first time the effect of anti-atherosclerotic drugs on plaque burden in progression-regression studies of coronary atherosclerosis [13,14,16,25].

At this regard, two IVUS imaging outcome measures were established: percent atheroma volume (PAV), calculated as the proportion of total vessel wall volume occupied by atherosclerotic plaque (plaque area divided by vessel area measured at the point of elastic lamina), and total atheroma volume (TAV), defined by the sum of atheroma areas measured in sequential frames. Prospective data demonstrated the correlation between PAV and patient at risk of future ischemic events [22,26]. A meta-analysis including 4137 patients from six different clinical trials showed that PAV, measured both at baseline and after 18–24 months of follow-up, is an independent predictors of major cardiovascular events [27].

More recently, the IVUS assessment has been improved with a new tool able to automatically assess lipid accumulation in coronary arteries: the NIRS. This method combines both IVUS and NIRS in a single multimodal catheter and, differently from IVUS, is able to quantify lipid component with high accuracy by detecting the spectroscopic signal of lipid molecules in the coronary artery wall [28].

Two different main imaging outcome measures have been described in NIRS studies to quantify intracoronary lipid accumulation [28,29]. The first one, known as *total lipid core burden index* (LCBI), is provided as the sum of the lipid signals along the interrogated vessel segment on a scale of 0 to 1000. The presence of high LCBI in a coronary artery not responsible for infarction at the time of imaging acquisition has been associated with a four times increased risk of developing future adverse cardiovascular events [29]. The second, most used NIRS outcome measure is the maximum LCBI in 4 mm of the investigated vessel (maxLCBI_4mm_). Several studies validated the maxLCBI_4mm_ as a reliable measure of vulnerability, showing the presence of a high maxLCBI_4mm_ (>400) in lesions responsible for MI [30] and in non-culprit lesions associated with future cardiovascular events [24,31]. Recently, in the international PROSPECT II study, the upper quartile of maxLCBI_4mm_ as detected by NIRS was used to define “lipid-rich” plaques, in addition to IVUS assessment, to identify vulnerable plaques responsible for coronary events at follow-up [23].

The OCT modality, developed in the late 1990s and first tested in the coronary vasculature in the early 2000s, uses near-infrared light waves to obtain high spatial and contrast resolution volumetric images. Among currently available ICI modalities, OCT has a resolution (axial 10–20 μm and lateral 20–90 μm) nearly 10 times greater than IVUS—at the expense of lower penetration depth (1–2 mm for OCT vs. 5–6 mm for IVUS) [20,32,33].

The detection of vulnerable plaque features by means of OCT was recently associated with a higher incidence of cardiovascular events at follow-up [21,34]. In the multicenter prospective CLIMA study enrolling 1003 patients with CAD, the presence of OCT-defined vulnerable plaque (thin fibrous cap <75 mm, MLA <3.5 mm^2^, large lipid arc >180° and macrophages) increased the risk of cardiac death and target vessel myocardial infarction by seven times [21], with the presence of a thin fibrous cap being the most important factor associated with clinical events in both women and men [35].

## 3. Pharmacological Treatment and Reduction in Plaque Progression

In the first studies on coronary angiography, LDL-C levels reduction of approximately 100 mg/dL with pravastatin and simvastatin was associated with the reduction in the progression of coronary artery disease, measured by the minimal luminal diameter [36,37]. As previously emphasized, the main limitation of these studies was the inability to evaluate atherosclerosis, as angiographic methodology only provides coronary luminal images.

In 1997, Takagi et al. [38] for the first time demonstrated a significant reduction in plaque progression as assessed by IVUS analysis in patients treated with 10 mg/day of pravastatin compared to the placebo group (−7% vs. +41%; *p* < 0.001).

Since then, several clinical trials have been conducted to investigate the effectiveness of lipid-lowering therapy in reducing the process of coronary atherosclerosis progression (Table 1). In the REVERSAL [13] study (Reversal of Atherosclerosis with Aggressive Lipid Lowering), Nissen et al. compared the effects of treatment with 80 mg/day of atorvastatin (intensive regimen) versus 40 mg/day of pravastatin (moderate regimen) in patients with documented coronary disease on angiography. The primary endpoint was PAV changing at follow-up. After 18 months of follow-up, patients undergoing the intensive regimen had significantly lower levels of LDL-C (79 mg/dL vs. 110 mg/dL, *p* < 0.01) compared to the moderate regimen group. Furthermore, patients treated with atorvastatin showed atheroma burden stabilization compared to baseline, differently from what observed in patients receiving pravastatin (−0.4% vs. 2.7%; *p* = 0.001). Simultaneously, both LDL-C (110 mg/dL vs. 79 mg/dL, *p* < 0.001) and C-reactive protein (CRP, −36.4% vs. −5.2%, *p* < 0.001) levels underwent a significantly greater reduction in the atorvastatin group [13]. Published in the same year of the REVERSAL trial, the ESTABLISH study had similar results, showing that early lipid-lowering therapy by atorvastatin 20 mg for 6 months significantly reduced the plaque volume in 70 patients with ACS [39].

## 4. Pharmacological Treatment and Plaque Regression

In 2006, the ASTEROID [16] study (A Study to Evaluate the Effect of Rosuvastatin on Intravascular Ultrasound-Derived Coronary Atheroma Burden) demonstrated for the first time the possibility for a lipid-lowering drug not only to decelerate the process of coronary atherosclerotic plaque progression but also to induce significant plaque regression. In this trial, the use of a high-dose statin, rosuvastatin 40 mg/day, showed a TAV reduction of over 6% and a PAV reduction of approximately 1% compared to the baseline. Overall, plaque regression was observed in 63.6% of patients treated with statin. Similarly, together with plaque regression, the therapy resulted in a significant reduction in LDL-C levels (−53%) and a significant increase in HDL-C levels (+15%). Other studies subsequently confirmed findings from ASTEROID. In the SATURN [14] (The Study of Coronary Atheroma by intravascular Ultrasound: the effect of Rosuvastatin vs. atorvastatin) trial two potent statins at maximum doses, rosuvastatin 40 mg/day and atorvastatin 80 mg/day, were compared. Both groups demonstrated significant regression in coronary atherosclerosis (Table 2). Specifically, a reduction in PAV was observed in 68.5% of patients treated with rosuvastatin and in 62.5% of patients in the atorvastatin group. Both PAV reduction (−1.22% vs. −0.99%, *p* = 0.17) and TAV reduction (−6.39 mm^3^ vs. −4.42 mm^3^, *p* = 0.01) were greater in rosuvastatin group, which also achieved lower LDL-C levels (62.5 mg/dL vs. 70.2 mg/dL, difference −7.5 mg/dL, *p* < 0.001) and higher HDL-C levels (48.6 mg/dL vs. 50.4 mg/dL, difference +1.8 mg/dL, *p* = 0.01). These data overall suggested no substantial differences in the two drugs in influencing plaque progression and regression mechanisms, as a consequence of the similar efficacy in reducing LDL-C levels.

The correlation between plaque regression and LDL-C values/inflammation markers emerged from early trials and was analysed in detail by Nicholls et al. in a meta-analysis published in JAMA in 2007 [48]. According to the authors, plaque regression occurred more frequently in patients with LDL cholesterol and C-reactive protein displaying a decrease of more than 29% and 30%, respectively [48].

In 2015, the PRECISE-IVUS [49] trial (Plaque Regression With Cholesterol Absorption Inhibitor or Synthesis Inhibitor Evaluated by Intravascular Ultrasound) evaluated the effects of combination therapy with ezetimibe and atorvastatin as compared to atorvastatin alone on plaque regression. The results showed that combination therapy, in addition to a greater reduction in LDL-C levels than the expected one (63.2 ± 16.3 mg/dL vs. 73.3 ± 20.3 mg/dL; *p* < 0.001), was associated with a higher prevalence of plaque regression, defined as a reduction in PAV (78% vs. 58%; *p* = 0.004) and TAV (75% vs. 58%; *p* = 0.02). Interestingly, a trial sub-analysis showed that plaque regression was more evident in patients with ACS as compared to patients with stable CAD [50]. These results confirm those observed in the JAPAN-ACS (Japan Assessment of Pitavastatin and Atorvastatin in Acute Coronary Syndrome) study, where statin therapy in ACS patients led to a significant reduction in plaque volume in non-culprit coronary arteries [51].

Regression studies investigating molecules different from statins led to controversial results. In the ILLUSTRATE trial [17], the use of Torcetrapid, a cholesterol ester transfer protein inhibitor able to increase HDL levels, was not associated with a reduction in atheroma volume. Similarly, the use of the Acyl-coenzyme A: cholesterol acyltransferase inhibitor (Pactimibe) in the ACTIVATE trial did not show any reduction in atheroma burden as compared to placebo [52,53]. It is interesting to note that both these drugs were not associated with an improved prognosis in patients with CAD [40].

On the other hand, in the CHERRY [43] trial (combination therapy of eicosapentaenoic acid and pitavastatin for coronary plaque regression evaluated by integrated backscatter intravascular ultrasonography) the use of eicosapentaenoic acid was associated with regression of the plaque lipid component and improved clinical outcomes [54]. The combination treatment of eicosapentaenoic acid and pitavastatin led to a greater reduction in lipid volume, measured by radiofrequency signal at integrated backscatter-IVUS, compared to statin therapy alone (63% vs. 45%, *p* = 0.048).

Of interest, women exhibited greater plaque volume reduction than compared with men in regression studies [55]. Recently, it has been hypothesized that this is due to greater condensed lipidic plaque features, despite smaller atheroma volume, in women compared with men [56].

In addition, a specific subgroup of patients who may benefit more from aggressive lipid lowering therapy is ACS patients. Indeed, patients with ACS have been described to harbour a more modifiable disease substrate which may benefit more from potent statin therapy compared with non-ACS patients [57].

As aforementioned, substantial data have shown that the use of monoclonal antibodies inhibiting PCSK9 has been able to further reduce LDL-C levels and improve clinical outcomes when added to statin therapy [11,12].

Published in 2016, the GLAGOV [42] trial (GLobal Assessment of Plaque ReGression with a PCSK9 AntibOdy as Measured by IntraVascular Ultrasound), was the first randomized, double-blind, placebo-controlled study designed to evaluate the effects of evolocumab on PAV in patients with angiographical documented CAD. In this trial, 988 patients undergoing optimized statin therapy were randomized to receive either subcutaneous evolocumab 420 mg once a month or placebo. After 78 weeks of treatment, mean LDL-C levels were significantly lower in the PCSK9 inhibitor group (36.6 vs. 93 mg/dL; difference, −56.5 mg/dL [95% CI, −59.7 to −53.4]; *p* < 0.001), with plaque regression occurring in over 60% of patients treated with evolocumab (compared to a percentage below 50% in the standard therapy group), and a greater reduction in both PAV (−0.95% vs. +0.05%, *p* < 0.001) and TAV (−5.8 mm^3^ vs. −0.9 mm^3^, *p* < 0.001).

Therefore, the addition of evolocumab vs. placebo to statin treatment in the GLAGOV trial resulted only in a 1% greater decrease in PAV. Thus, the extent of atheroma volume reduction in the GLAGOV trial appeared modest in relation to the net clinical benefit showed in outcomes clinical trials achieving similar on-treatment LDL-C levels [11,12].

## 5. Pharmacological Treatment and Plaque Stabilization

Can such a modest reduction in atherosclerotic plaque (1% PAV reduction) explain the great clinical benefit achieved with high-intensity lipid-lowering therapy? The following years answered this question thanks to the introduction of new methods, such as NIRS and OCT, able to detect favourable changes in coronary atheroma composition and microstructure, i.e., plaque stabilization (Table 2).

The Yellow study [41], (Reduction in Yellow Plaque by Aggressive Lipid-Lowering Therapy) published in 2013, analysed significant coronary lesions using NIRS-IVUS technique at baseline and after 7 weeks of therapy with rosuvastatin 40 mg/day. The study demonstrated that short-term aggressive statin therapy can modify both the quantity and the composition of coronary atherosclerotic plaques by reducing their lipid content. The median percentage reduction in maxLCBI_4mm_ was significant in subjects treated with rosuvastatin as compared to those on standard therapy (32% vs. 0.6%, *p* = 0.02). Similarly, the EASY-FIT trial involving 70 patients with ACS, treatment with atorvastatin 20 mg/day resulted in plaque stabilization as detected by an increasing in OCT-detected fibrous cap thickness higher than that achieved with 5 mg/day atorvastatin [44].

Consistent with these findings, an observational study on 53 patients showed a reduction in maxLCBI_4mm_ by NIRS-IVUS in patients treated with PCSK9 inhibitors as compared to those on statin monotherapy [58].

In the IBIS-4 (Integrated Biomarker Imaging Study-4) study, 103 patients underwent IVUS and OCT of two non-infarct-related coronary arteries in the acute phase of STEMI. At 13 months follow-up, the therapeutic regimen with high-dose rosuvastatin was demonstrated to promote plaque stabilization by increasing OCT-derived fibrous cap thickness by +24.4 μm (*p* = 0.008) and by reducing macrophage accumulation [45]. A recent randomized trial [46], including 48 patients, showed a reduction in lipid index and macrophages, and an increase in fibrous cap thickness evaluated by OCT in patients treated with alirocumab compared to those on statins. Similarly, in the OCTIVUS sub-study involving 87 statin-naïve STEMI patients, aggressive treatment with ezetimibe and atorvastatin 80 mg showed further changes in plaque composition evaluated by OCT, including an increase in fibrous cap thickness and a reduction in lipid content and macrophage infiltration [59].

The reduction in macrophage infiltration can also represent one of the pathophysiological aspects behind the beneficial effect of high-intensity lipid lowering therapy in CAD patients. In agreement with this hypothesis, the presence of a large and superficial macrophage accumulation at OCT has been recently associated with a higher incidence of adverse events at follow-up, especially in presence of high CRP levels [60,61].

Although the findings are relevant, the above-mentioned studies on plaque stabilization did not perform any power calculations, and the small number of patients was a limitation for results interpretation. The first data with adequate sample size and statistical power came from two landmark randomized trials: the HUYGENS [47] and the PACMAN-AMI trial [18]. Both trials compared the results of PCSK9 inhibitor therapy vs. placebo in patients treated with high-intensity statin lowering therapy.

The primary efficacy endpoint in the HUYGENS [47] trial was the absolute change in minimum fibrous cap thickness from baseline to week 50 measured by serial OCT. Secondary efficacy measures included the percentage change in minimum fibrous cap thickness and nominal changes in minimum mean fibrous cap thickness of all images, lipid arc, and lipid content length. Among the 135 patients with evaluable images at follow-up, the evolocumab group achieved significantly lower mean LDL-C levels (28.1 vs. 87.2 mg/dL; *p* < 0.001). The evolocumab group showed a greater increase in minimum fibrous cap thickness (FCT) (+42.7 vs. +21.5 μm; *p* = 0.015), a reduction in maximum lipid arc (−57.5° vs. −31.4°; *p* = 0.04), and a reduction in macrophage index (−3.17 vs. −1.45 mm; *p* = 0.04) in plaques with a lipid component. Additionally, a greater regression in the percentage of atheroma volume with evolocumab compared to placebo plus optimal medical therapy (−2.29% ± 0.47% vs. −0.61% ± 0.46%; *p* = 0.009) was observed.

In the PACMAN-AMI trial [18], non-culprit coronary arteries of 300 patients were studied using intravascular ultrasound (IVUS), near-infrared spectroscopy (NIRS), and optical coherence tomography (OCT) at admission and after 52 weeks. Compared to the group receiving high-intensity statin therapy alone, patients treated with alirocumab and statins showed a greater reduction in the average percentage change of PAV in non-culprit arteries (primary efficacy endpoint) (−2.13% vs. −0.92%; *p* < 0.001). Moreover, the addition of alirocumab was associated with a greater reduction in plaque lipid component (the mean variation of maxLCBI_4mm_ was −79.42 with alirocumab and −37.60 with placebo, *p* = 0.006), a greater increase in minimum fibrous cap thickness (mean change 62.67 μm vs. 33.19 μm, *p* = 0.001), and a greater reduction in the angular extent of macrophage infiltration (*p* < 0.001). Interestingly, the greater reduction in the angular extent of macrophage infiltration observed in the PACMAN-AMI study was not associated with a significant reduction in high-sensitivity C-reactive protein levels, differently from studies using statins. However, the absence of correlation between local and systemic markers of inflammation has been previously reported by pre-clinical and clinical studies [61,62].

The consistency between lipid component reduction and increase in cap thickness in plaque stabilization studies is not surprising. In fact, recent studies reported that the presence of a thin fibrous cap is significantly higher in lesions with large NIRS-derived lipid accumulation than in non-lipid-rich lesions [63,64].

## 6. Future Perspectives

Despite the high accuracy, the invasive nature of the above-described techniques represents the main limitation to their use in routine clinical practice. In recent years, several non-invasive techniques have been developed in order to achieve similar results.

Coronary computed tomography angiography (CCTA) is now a clinically established imaging technique, allowing non-invasive identification and characterization of coronary atherosclerotic disease [65]. CCTA can not only detect calcified plaques but also identify the so-called adverse plaque characteristics (APC), as well as low-attenuation plaques and positive remodelling [65]. CCTA calculated plaque volume and APC have been associated with the presence of lesion-related ischemia [66,67]. In addition, recent studies have demonstrated the ability of CCTA to quantify plaque burden, showing good correlation with IVUS images [68]. High-risk plaques with CCTA positive remodelling and low attenuation have been associated with thin fibrous caps and macrophage infiltration at OCT evaluation [69]. New evidence about the use of CCTA in plaque regression/stabilization studies are expected in the near future, such as the ongoing GOLDILOX-TIMI 69 study (clinicaltrials.gov ID: NCT04610892).

Cardiac magnetic resonance (CMR) and positron emission tomography (PET) are other emerging modalities to non-invasively detect coronary atherosclerotic features [70].

CMR enables the visualization of the coronary lumen and characterization of its wall [70,71,72]. In the Multi-Ethnic Study of Atherosclerosis (MESA), CMR demonstrated that patients with more cardiovascular risk factors have a significantly increased coronary wall thickness [72]. CMR can also visualize the positive vessel remodeling [73]. However, coronary plaque regression using magnetic resonance imaging has not been investigated yet. The sub-optimal resolution of the technique and the coronary vessels motion currently represent important limitations for its clinical applications.

Positron emission tomography (PET) is an imaging method capable of detecting and quantifying pathophysiological processes associated with atherogenesis and inflammation [74]. 18F-fluorodeoxyglucose (FDG) is the most commonly used radioligand in PET imaging for atherosclerosis studies. Originally used for cancer staging, the incidental finding of FDG accumulation in arterial territories has revealed to be useful in detecting and quantifying inflammation within the atheroma [75]. FDG uptake seems to identify non-stenotic symptomatic carotid plaques on high-resolution magnetic resonance imaging, supporting the concept that the severity of stenosis does not represent the only disease severity marker [76]. Inflammation, assessed by both FDG uptake and histology, is increased in plaques with high-risk morphological features [77].

Furthermore, non-invasive studies will also help address unmet needs, such as serial evaluations at different time intervals to investigate the perfect start of a potent lipid-lowering therapy and the legacy effect of it after its discontinuation.

## 7. Conclusions

Recently, new pharmacological therapies have significantly improved the prognosis of patients with coronary atherosclerotic disease. Invasive imaging studies helped to better understand many pathophysiological aspects, and serial trials have demonstrated that more aggressive pharmacological treatments can even determine plaque regression and stabilization. In the future, new data will be required to fully understand the impact of these findings in clinical practice and develop new non-invasive techniques to detect coronary plaque features.

## Figures and Tables

**Table 1:** Prospective and randomized trials on coronary atherosclerotic plaque regression using intravascular ultrasound (IVUS).

Trial/Author	Year	Trial Design	Therapy	Patients (N)	Population	Age (y) Active Drug vs. Placebo	Women (%)	Follow-Up (Months)	Mean Change in PAV (%)	Mean Change in TAV (%)
**Takagi et al. [** 38 **]**	1997	R	Pravastatin 10 mg vs. control	25	Patients undergoing PCI, TOT-C between 200 and 260 mg/dL	56 vs. 56	0	36	−7 vs. +27 (area) *p* < 0.0005	−7 vs. +41 (area) *p* < 0.0005
**REVERSAL [** 13 **]**	2004	R	Atorvastatin 80 mg vs. Pravastatin 40 mg	502	Documented coronary artery disease (at least one stenosis ≥20%; target segment with stenosis ≤50% and minimum length 30 mm)	55.8 vs. 56.6	29 vs. 27	18	+0.2 vs. +1.6 *p* < 0.001	−0.9 vs. +4.4 *p* = 0.02
**ASTEROID [** 16 **]**	2006	P	Rosuvastatin 40 mg	349	Documented coronary artery disease (at least one stenosis ≥20%; target segment with stenosis ≤50% and minimum length 40 mm)	58.5	29.8	24	−0.79% *p* < 0.01	−6.8% *p* < 0.01
**ACTIVATE [** 40 **]**	2006	R	Pactimibe 100 mg vs. placebo	408	Documented coronary artery disease (at least one stenosis ≥20%; target segment with stenosis ≤50% and minimum length 30 mm)	58.8 vs. 59.6	34.2 s 28.4	18	+0.69% vs. +0.59% *p* = 0.77	−1.3 mm^3^ vs. −5.6 mm^3^ *p* = 0.03
**ILLUSTRATE [** 17 **]**	2007	R	Atorvastatin vs. Atorvastatin/Torcetrapib 60 mg	1188	Documented coronary artery disease (at least one stenosis ≥ 20%; target segment with stenosis ≤ 50% and minimum length 40 mm)	57 vs. 56.9	29.5 vs. 29.6	24	+0.19% vs. + 0.12% *p* = 0.72	−6.3 mm^3^ vs. −9.4 mm^3^ *p* = 0.02
**SATURN [** 14 **]**	2011	R	Atorvastatin 80 mg vs. Rosuvastatin 40 mg	1039	Documented coronary artery disease (at least one vessel with stenosis > 20%; target segment with stenosis ≤50%)	57.4 vs. 57.9	25.6 vs. 27.1	8.7	−0.99% vs. −1.22% *p* = 0.17	−4.42 mm^3^ vs. −6.39 mm^3^ *p* = 0.01
**YELLOW [** 41 **]**	2013	R	Rosuvastatin 40 mg vs. standard therapy	87	Multivessel stable coronary artery disease (at least two vessels with stenosis ≥70%)	64.4 vs. 62.9	20.5 vs. 27.9	1.7	-	-
**GLAGOV [** 42 **]**	2016	R	Evolocumab 420 mg once at month vs. placebo	968	Documented coronary artery disease (at least one stenosis ≥20%; target segment with stenosis ≤50%) on optimized statin therapy	59.8 vs. 59.8	27.9 vs. 27.7	19	−0.95% vs. + 0.05% *p* < 0.001	−5.80 mm^3^ vs. −0.91 mm^3^ *p* < 0.001
**CHERRY [** 43 **]**	2017	R	Pitavastatin 4 mg/EPA 1800 mg vs. Pitavastatin 4 mg	193	Patients undergoing PCI	67 vs. 68	20 vs. 16	8	-	-

**Table 2:** Randomized trials on coronary plaque stabilization using OCT and NIRS-IVUS.

Trial/Author	Year	Patients (n)	Population	Mean Age (Years)	Women (%)	Therapy	Dose	Follow-Up (Months)	Mean Change in Fibrous Cap Thickness	Mean Change in Lipid Burden	Mean Change in Minimal Luminal Area	Macrophage Accumulation
**EASY-FIT [44]**	2014	60	Non treated unstable angina and dislipidemia	63 vs. 69	13 vs. 27	Atorvastatin	20 mg vs. 5 mg	12	+73 µm (*p* < 0.001) vs. +19 µm (*p* = 0.002)	−50° (*p* < 0.001) vs. −20° (*p* < 0.001)	−0.05 mm^2^ (*p* = 0.256) vs. −0.09 mm^2^ (*p* = 0.101)	−4.5 (*p* < 0.001) vs. −2 (*p* < 0.001) (accumulation grades)
**IBIS-4 [45]**	2015	103	ACS (STEMI)	58.2	9.7	Rosuvastatin	40 mg	13	+23 µm *p* = 0.008	−12.4°	-	−3.2° (angolar extension) *p* < 0.0001
**ALTAIR [46]**	2019	24	ACS or stable CAD	61.3 vs. 61.3	33.3 vs. 25.8	Alirocumab + rosuvastatin vs. rosuvastatin alone	75 mg every 2 weeks	36 weeks	+18 µm vs. +13.2 µm *p* = 0.029	−15.1° vs. −8.4° *p* = 0.008	+0.20 mm^2^ vs. +0.13 mm^2^ *p* = 0.006	-
**HUYGENS [47]**	2021	161	ACS (NSTEMI)	60.5	28.6	Evolocumab vs. placebo	420 mg/month	52 weeks	+42.7 µm vs. +21.5 µm *p* = 0.015	−57.5° vs. −31.4° *p* = 0.04	-	−3.17 mm vs. −1.45 mm (macrophages index) *p* = 0.04
**PACMAN [18]**.	2022	300	ACS (NSTEMI or STEMI)	58.5	18.7	Alirocumab vs. placebo (together with Rosuvastatina)	150 mg every 2 weeks	12	+62.67 µm vs. +33.19 µm *p* = 0.001	−79.42 vs. −37.60 (LCBI) *p* = 0.006	-	−25.98° vs. −15.95° (angular extension)

## Data Availability

Not applicable.

## Abbreviations

ACS	acute coronary syndromes
APC	adverse plaque characteristics
CAD	coronary artery disease
CCTA	computed tomography angiography
CMR	Cardiac magnetic resonance
CRP	C-reactive protein
FDG	18F-fluorodeoxyglucose
ICI	intracoronary imaging
LCBI	lipid core burden index
LDL-C	low-density lipoprotein cholesterol
MaxLCBI_4mm_	maximum LCBI in 4 mm of the investigated vessel
MI	Myocardial infarction
IVUS	intravascular ultrasound
NIRS	new near-infrared spectroscopy
OCT	optical coherence tomography
PCSK9	proprotein convertase subtilisin kexin type 9
PET	positron emission tomography
